# Annotations

**Published:** 1909-08-14

**Authors:** 


### ANNOTATIONS.

### Fighting Tuberculosis.

We have already on more than one occasion referred to the excellent work that is being done by the National Association for the Prevention of Consumption and other forms of Tuberculosis, and it is with great pleasure that we draw attention to the continued popularity of the Exhibition recently organised under its auspices. It will be remembered that this display was at first housed in the Art Gallery Whitechapel, where, during a fortnight, it was visited by no fewer than 72,000 people. It is now domiciled at the Imperial International Exhibition (White City), Shepherd's Bush, and was formally opened there on Friday, August 6, by Lord Balfour of Burleigh. In his opening remarks Lord Balfour laid stress on the educational importance and value of the Exhibition, and every member of the profession will cordially endorse his remarks. Such popular displays are of undoubted value, especially when they are so thoroughly organised as this one is, and when the lessons they are designed to teach are so urgently needed by the people. Perhaps on no other subject is public ignorance—we are almost tempted to write public prejudice—so rampant as on this socially and nationally important question of tuberculosis, and it is urgent that the population of our large cities, at least, should be shown plainly and graphically the true state of the case against consumption. This the Association is doing ably and well, and it is gratifying to find that its efforts are meeting with the appreciation they so richly deserve. It has already received enough invitations to show the Exhibition in the various districts of London and the provincial cities to keep it occupied for more than a year. In congratulating the Association on this well merited success of its endeavours in its crusade against a national scourge, we would venture to hope that means might be found for establishing the Exhibition on a permanent basis in London.

### Cancer Research.

The late Mr. Harry Barnato left by will a sum of £250,000 for the purpose of founding some charitable institution in commemoration of his brother, Mr. Barney Barnato, and his nephew, Mr. Woolf Joel, both of whom predeceased him. Mr. Barnato's trustees have now decided, after a very full and careful consideration of the various schemes which have been submitted to them, to apply this money in building, equipping, and endowing an institution for the reception of patients suffering from cancer, a suitable site in Nassau Street, adjoining the Middlesex Hospital especial cancer wards has been procured, and no time will be lost in carrying out the scheme. We understand that the new institution is to be administered, except as regards its finance, in connection with the Middlesex Hospital. We congratulate that institution upon the splendid gift which will certainly, as Mr. Bland Sutton expresses it in the *Times* of Monday last, change for the better the conditions under which the Cancer Investigation Committee has been working during the last few years. Hitherto the committee has been handicapped not only by lack of funds, but by other disadvantages, and there has been scant opportunity of acquiring the services of highly-skilled specialists to carry out the necessary work. Continental laboratories which devote attention to this difficult and interesting problem have been on a better footing, as in most instances they have State or communal support. The English Committee has done yeoman service, and is likely to do much more important work in the future now that this bequest enables them to labour without being stinted for the necessary funds to carry on their investigations.

### The Recent Outbreak of Plague in West Africa.

Some interesting points regarding the recent outbreak of plague on the West African Gold Coast are presented in Prof. W. J. Simpson's lately published report. The epidemic, it appears, was a large one, no fewer than 344 cases of the disease occurring, 300 of these ending fatally. "In the first outbreak, from January 5 to April 10, there were 302 cases of plague of which 258 proved fatal. The outbreak in Accra was mainly bubonic and associated with a plague epizootic among rats, but the outbreak in the villages was, with the exception of three cases, entirely pneumonic, and with no mortality or infection of rats. In the second outbreak, from early in May to June 26, there were thirty-five cases of pneumonic plague in the colony, all of which were fatal. There was no rat infection connected with any of these. In the third outbreak, from July 28 to August 17, there were six cases of plague in Accra, all pneumonic and fatal." Over 35,000 preventive inoculations were carried out. Only four inoculated persons were attacked with the disease, all pneumonic; one of these recovered, the others died. Prof. Simpson believes that he frequently observed the arrest of the disease in localities and villages after all the inhabitants were inoculated, even though the pneumonic type of the disease prevailed. The great prevalence of pneumonic cases is interesting, and Prof. Simpson and the other authorities must be warmly congratulated on getting the upper hand of such a widespread infection so rapidly. Rats examined in the laboratory showed two species—(*a*) Cricetomys Gambiensis (waterhouse) and (*b*) Mus decumanus. No specimens of Mus rattus were brought to the laboratory. Dr. Graham, who examined these rodents, found a great variety of parasites on them, and Prof. Simpson thinks the find is important in that the flea alone has hitherto occupied sole attention as the only parasite possibly conveying infection. The disease did not spread to any of the other colonies on the West Coast, which is highly satisfactory. As to how the Gold Coast became infected there does not seem to be any definite evidence, but the importation might, of course, have taken place by rats from ships or steamers calling there. The epidemic may really be a blessing in disguise, as it has called attention to the highly insanitary conditions prevailing in Accra and other West Coast towns, and now it is to be hoped that the Government will see their way, eventually at least, to remedy some of these evils.

